# Tryptophan, kynurenine pathway, and diabetic ketoacidosis in type 1 diabetes

**DOI:** 10.1371/journal.pone.0254116

**Published:** 2021-07-19

**Authors:** William H. Hoffman, Stephen A. Whelan, Norman Lee

**Affiliations:** 1 Department of Pediatrics, Medical College of Georgia, Augusta University, Augusta, Georgia, United States of America; 2 Department of Chemistry, Chemical Instrumentation Center (CIC), Boston University, Boston Massachusetts, United States of America; University of Colorado Denver School of Medicine, UNITED STATES

## Abstract

Diabetic ketoacidosis (DKA) is a serious complication of complete insulin deficiency and insulin resistance in Type 1 diabetes (T1D). This results in the body producing high levels of serum ketones in an attempt to compensate for the insulin deficiency and decreased glucose utilization. DKA’s metabolic and immunologic dysregulation results in gradual increase of systemic and cerebral oxidative stress, along with low grade systemic and cerebral inflammation and the development of pretreatment subclinical BE. During treatment the early progression of oxidative stress and inflammation is hypothesized to advance the possibility of occurrence of crisis of clinical brain edema (BE), which is the most important cause of morbidity and mortality in pediatric DKA. Longitudinal neurocognitive studies after DKA treatment show progressive and latent deficits of cognition and emphasize the need for more effective DKA treatment of this long-standing conundrum of clinical BE, in the presence of systemic osmotic dehydration, metabolic acidosis and immune dysregulation. Candidate biomarkers of several systemic and neuroinflammatory pathways prior to treatment also progress during treatment, such as the neurotoxic and neuroprotective molecules in the well-recognized tryptophan (TRP)/kynurenine pathway (KP) that have not been investigated in DKA. We used LC-MS/MS targeted mass spectrometry analysis to determine the presence and initiation of the TRP/KP at three time points: A) 6–12 hours after initiation of treatment; B) 2 weeks; and C) 3 months following DKA treatment to determine if they might be involved in the pathogenesis of the acute vasogenic complication of DKA/BE. The Trp/KP metabolites TRP, KYN, quinolinic acid (QA), xanthurnenic acid (XA), and picolinic acid (PA) followed a similar pattern of lower levels in early treatment, with subsequent increases. Time point A compared to Time points B and C were similar to the pattern of sRAGE, lactate and pyruvic acid. The serotonin/melatonin metabolites also followed a similar pattern of lower quantities at the early stages of treatment compared to 3 months after treatment. In addition, glutamate, n-acetylglutamate, glutamine, and taurine were all lower at early treatment compared to 3 months, while the ketones 3-hydroxybutaric acid and acetoacetate were significantly higher in the early treatment compared to 3 months. The two major fat metabolites, L-carnitine and acetyl-L-carnitine (ALC) changed inversely, with ALC significantly decreasing at 2 weeks and 3 months compared to the early stages of treatment. Both anthranilic acid (AA) and 3-OH-anthranilic acid (3OH-AA) had overall higher levels in the early stages of treatment (A) compared to Time points (B and C). Interestingly, the levels of AA and 3OH-AA early in treatment were higher in Caucasian females compared to African American females. There were also differences in the metabolite levels of QA and kynurenic acid (KA) between genders and between races that may be important for further development of custom targeted treatments. We hypothesize that the TRP/KP, along with the other inflammatory pathways, is an active participant in the metabolic and immunologic pathogenesis of DKA’s acute and chronic insults.

## Introduction

Diabetic ketoacidosis (DKA) in type 1 diabetes (T1D) is an acute, complicated, metabolic/immunologic condition that involves: dehydration [1]; transient hypertension [2, 3]; oxidative stress [4, 5]; and numerous recognizable metabolic [6–12] and immunologic dysregulations [13–16], in addition to a prothrombotic state [17]. The oxidative and inflammatory stresses of the hyperosmolar, acidotic milieu can cause recognized [18] and unrecognized interactions of dysregulated molecules [19, 20], resulting in perturbations of various degrees to the paracellular and cellular components of the blood-brain barrier (BBB) and the brain [21, 22].

While the research is limited, we believe that early inflammatory DKA pathways, prior to and during treatment, are important candidates for involvement in the pathogenesis of BE/DKA, a life-threatening insult for children with T1D/DKA. An additional candidate for inflammation is the kynurenine pathway (KP), with the newly formed tryptophan (TRP)/kynurenine (KP) metabolites that are likely initiated prior to DKA treatment. This activation can result in dysregulation or over activation with production of both neurotoxic and neuroprotective molecules. In addition to the molecules of the kynurenine pathway (KP) and the immune system [23], the KP also connects the innate and adaptive immune systems [24], advancing the severely dysregulated milieu. The complexity and progression of the dysregulation increases the potential value of early study and biomarker identification [25] for studying the pathogenesis of the neurological crisis, and the goal of identifying potential metabolic as well as immunological molecular target(s) for intervention.

Acute clinical BE in DKA is an uncommon crisis [26] in contrast to the frequent development of subclinical BE [27, 28] and pulmonary edema [29], that occur prior to treatment. Until recently, severe DKA and subclinical BE were thought to resolve without significant sequelae. However, the subclinical neurocognitive studies [30, 31] have increased the sense of urgency for more effective DKA treatment.

Timed, systemic, longitudinal KP biomarkers of cerebral capillary perturbations, when KP molecular modulations are common, increase the candidates that also result in possible transient injury to other organs, such as the myocardium [3, 32]. The kynurenine pathway (KP) metabolites and their modulations have numerous targets via blocking; noxious or non-noxious stress; and oxidative stress, inflammation and immunity. These KP molecules disrupt physiological pathways and cause toxic cellular damage [33] that can be heightened by stress levels of glucocorticoids [34].

A NEJM editorial pointed out [35] that research on the pathogenesis of BE/DKA has focused for an extended time on dysregulation of fluids and electrolytes (initially Dillon, 1936), and encouraged that additional considerations be given to this metabolic crisis. Liu, et al. [36] recently called attention to the growing importance of the metabolic KP and its generation of cellular energy homeostasis. Yet KP intermediates can also impair energy metabolism with a broad range of targets and effects [37–39].

KP’s initial, rate-limiting, inflammatory/immunomodulatory catabolic enzyme (indoleamine 2,3-dioxygenase [IDO]) and the two KPs result from TRP degradation, and have distinct enzymes and subsequent interactions between the contrasting neuroactive metabolites (neuroprotective and neurotoxic). Numerous enzymes are synthesized in macrophages/microglia, astrocytes, and neurons [40]. The metabolic neurotoxic quinolinic acid (QA) has limited constitutional synthesis in the brain, with its primary site of synthesis being the liver, then passing slowly through the BBB. The slow BBB passage of QA is in keeping with an important, likely extended, early period of slowly progressive vasogenic perturbations/alterations of the integrity and cohesion of the BBB [15, 16, 27] prior to the clinical crisis of BE. QA is also an agonist for activation of glutamate excitotoxicity via N-methyl-D-aspartate (NMDA)–a multifunctional ionotropic cell membrane receptor complex that responds to ligand binding by opening ion channels into cells that increases oxidative stress. NMDA also modulates inflammation, immunoregulation and the release of arachidonic acid [41]. KYN acid (KYNA), a modulator of QA results in NMDA’s attenuated inflammation.

Our objective was to determine the presence and initiation of the KP in DKA’s metabolic and immunologic crisis, and whether it has a potential role in the pathogenesis of the acute vasogenic complication of DKA/BE [6–17] by considering: a) KP’s time proximity to the transient TRP depletion; b) the transition to the formation of kynurenine metabolites and the metabolic/inflammatory cascade relative to the SIR of DKA; and c) whether these KP metabolites might serve as biomarkers of progressing subclinical BE [42, 43].

Finally, we want to emphasize that acute and more protracted insults can begin early in a DKA crisis, even with the onset of DKA. We assayed the KP metabolites that have not previously been reported, during and after the treatment of uncomplicated, severe T1D/DKA.

## Materials and methods

Fifteen of the original 17 patients [3] were included in this study. Two of the initial cases had no plasma remaining and two had only two of the three samples. Thus a total of 43 samples were assayed for the major tryptophan/kynurenine pathway metabolites (e.g. 3-OH-anthranilic acid, anthranilic acid, kynurenic acid, kynurenine, picolinic acid, quinolinic acid, tryptophan, and xanthurenic acid), as well as other metabolites mentioned in this manuscript. We arbitrarily chose 3 months post DKA treatment with a history of no ketonuria for the 2 weeks prior to a routine clinic visit for the (C) samples (second baseline post DKA) to be obtained.

A prospective longitudinal design was utilized to study a cohort of children and adolescents with uncomplicated T1D/DKA for evidence of inflammation ie. TRP/KYN pathway. The study received Expedited Approval by the IRB at East Carolina University (ECU) Brody School of Medicine since blood samples were only obtained at the time of routine blood sampling for the treatment of DKA and at follow up visits. The study was conducted in accordance with the Declaration of Helsinki. A total of fifteen children and adolescents between the ages of 9.5 and 17 years presenting with DKA (total CO2 = /< 12 mmol/L) were invited to enroll in the study. Informed consent was signed by the legal guardian and assent obtained from patients 9 years and over when not prohibited by the severity of illness. In such cases, patient assent was obtained when clinical improvement permitted. Patients referred from outlying hospitals were stabilized prior to being transported to ECU after consultation with the accepting attending physician in the Pediatric Intensive Care Unit. Treatment was according to previously published guidelines [44] with each patient serving as their own control at (2 weeks (B) and 3 months (C) and as the baseline. Transfer of children and adolescents for DKA treatment was routine in this part of North Carolina at the time of the study.

### Study evaluation and analysis

Pretreatment values were obtained for blood pressure (BP), heart rate (HR), complete blood count (CBC), glucose (BG), electrolytes, urea nitrogen (BUN) and creatinine at the referring hospitals. The start of treatment was defined as the initiation of continuous intravenous insulin. In addition to the pretreatment BP, BPs were also obtained and recorded hourly with an automated oscillometric device and appropriately sized BP cuff. BPs were also obtained hourly after initiation of insulin treatment between 6–12 hours (A); 2 weeks post correction (B); and baseline, 3 months post discharge (C). The samples were for tryptophan/kynurenine pathway metabolites as well as others mentioned throughout this study. Capillary BGs were obtained hourly, electrolytes, and BUN were measured every two to four hours with the initial sample (A) obtained on admission. At each of the routine chemistry collections a sample of 7 cc was also obtained for the study. A CBC and differential was repeated at 24 hours, post initiation of IV insulin. The 6–12 hrs sampling interval for (A) was a logistical consideration because differing distances and means of transfer and the time range that we have found as the most inclusive time for the SIR peak. No IV vitamins were given during treatment.

None of the patients were known to have hypertension, diabetic retinopathy, nephropathy or coronary artery disease. Exclusion criteria included a history or physical findings suggestive of an acute or chronic infection, emotional or physical disability or autoimmune conditions other than chronic lymphocytic thyroiditis.

### Metabolite extraction

Plasma 50 μL is added to an Eppendorf tube and precipitate solution (8:1:1 Acetonitrile: Methanol: Acetone) was added to make a solution of 1:8 (sample: solvent) ratio, vortexed sample to ensure mixing and sample kept on ice for 30 minutes to further precipitate proteins. Samples are then centrifuged at 15,000 rcf for 10 mins at <10°C to pellet proteins. Supernatant was transferred to new, labeled tube making sure to leave behind protein pellet. Sample was dried in Speed Vacuum Centrifuge and then reconstituted by adding 50μl μL H_2_0 with 0.1% formic acid and vortexed. Samples are again placed on ice for 15 minutes, centrifuged again to remove any protein or lipid that was not removed. Supernatant was transferred to labeled, glass LC vial with glass insert. Samples were then placed into Agilent HPLC 1100 series auto sampler.

### LC-MS/MS

An Agilent HPLC 1100 series was used with a Waters Acquity CSH^™^ Phenyl-Hexyl 1.7μM 2.1 x 50mm column. A Sciex API 4000 triple quadrupole mass spectrometer with an ESI source was used in positive mode with first scan event a full MS scan at 55.0-1000m/z. For positive polarity detection of targeted tryptophan/kynurenine pathway metabolites, as well as other metabolites a gradient of 95% buffer A was set at 0.00–1.00min, buffer B increased to 15% at 4.00min, then buffer B increased to 95% to 7.00, maintained to 8.00 min then decreased to 5% buffer B at 8.50min through 10.0min. For negative polarity detection of metabolites a gradient of 95% buffer A (H2O and 0.1% formic acid) and 5% buffer B (methanol) at 0–0.5min, increased buffer B to 95% at 5min, increased buffer B to 98% at 8.5min and then decreased buffer B to 5% to 9.0–10.0min at a flow rate of 0.15ml/min. In negative polarity mode for analysis similar parameters were set at 55.0–1000 m/z.

### Statistical analysis

T-Test was performed on the metabolites using Microsoft excel software comparing time points A to B, A to C and B and C where indicated using two tailed distributions assuming unequal variances and within-subjects (Supporting information).

## Results and discussion

This study confirms and extends that TRP/KP is another important pathway of inflammation in the severely dysregulated metabolic and immunologic milieu of DKA. The timing of the early inflammatory surge in DKA that involves the SIR [11] includes the KP. This complexity is supported by other systemic pretreatment longitudinal DKA inflammatory studies that include: TRP depletion [12]; the SIR [11, 13]; alpha dicarbonyls/AGE-RAGE [8–10]; and the complement cascade [14]. Our study indicates KP is an early participant in the inflammation/perturbation of DKA and likely begins prior to treatment. While IDO, the initial metabolic enzyme, is cited to be initiated by one of the SIR’s inflammatory cytokines [11, 12] it remains to be determined if beta hydroxybutyrate (BOHB) [45] is also an early in vivo initiator of IDO and that increases KA formation. The ketone body BOHB has the greatest systemic increase during the lipolysis of DKA’s advancing insulin-deficient state, and increases the brain’s KYNA synthesis and with has the ability to modulate. Ketone bodies are frequently characterized as neuroprotective effects [46]; however, these metabolites are also activators of cerebral capillary endothelial cells (CCEC) [47–49].

**Fig 1:**
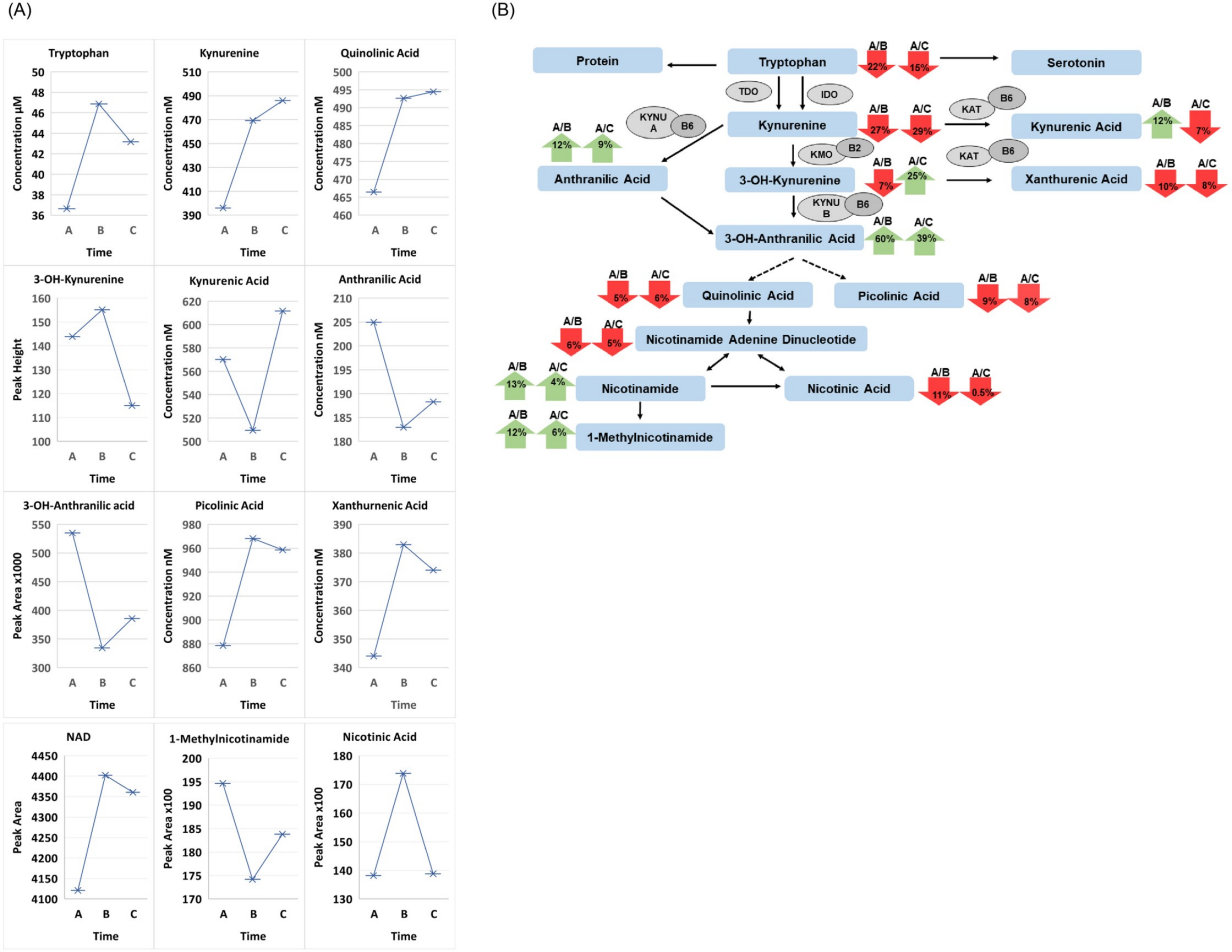
**A**. Comparison of treatment for all patients over 3 time points for the metabolites Time A at 6-12hours, Time B at 2 weeks and Time C at 3 months between all patients for tryptophan, kyunrenine, quinolinic Acid (QA), kynurenic acid (KA), 3-OH-anthranilic acid, anthranilic acid, picolinic acid, xanthurenic acid, nicotinamide adenine dinucleotide (NAD), 1-methylnicotinamide and nicotinic Acid. **B**. Flow diagram of tryptophan/kynurenine pathway. Pathway displaying the percent change differences between Time A divided by Time B and Time A divided by Time C. Red indicates a decrease while green indicates an increase. Major enzymes and cofactors in the Trp/KP: indoleamine 2,3-dioxygenase (IDO); tryptophan-2,3-dioxygenase (TDO); kynurenine aminotransferase (KAT); and kynurenine 3-monooxygenase (KMO); L-kynureninas A and B (KYNU A and KYNU B); vitamin B6; and vitamin B2.

**Fig 2:**
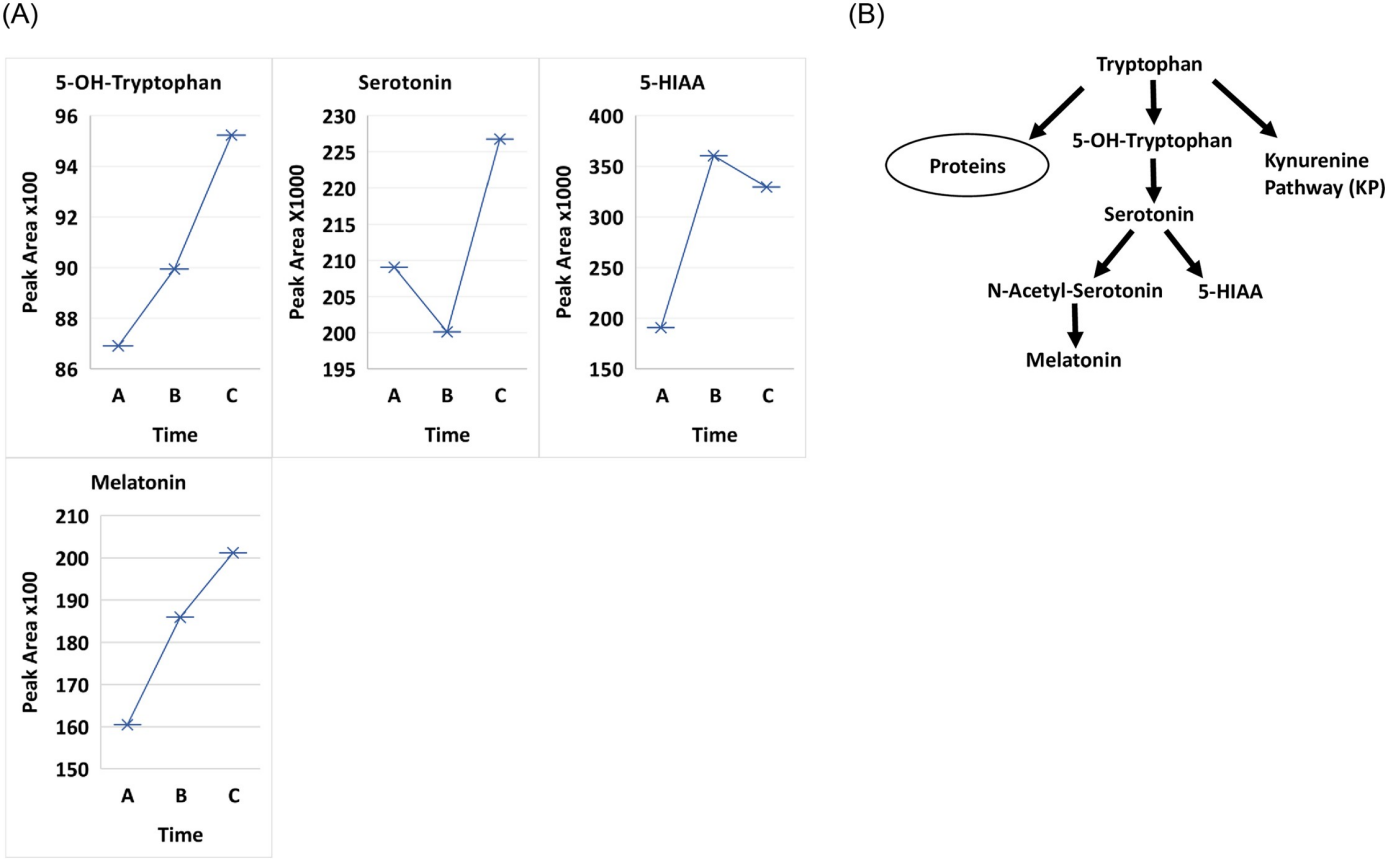
**A**. Comparison of treatment over 3 time points Time A at 6-12hours, Time B at 2 weeks and Time C at 3 months between all patients for metabolites 5-hydoxytryptophan, serotonin, 5-hydroxyindoleacetic acid (5-HIAA) and melatonin. **B**. Flow diagram of serotonin/melatonin pathway.

Eleven of 15 patients in our study had their lowest TRP concentrations at 6–12 hours (A). The average TRP concentration at (A) was decreased from the baseline concentrations at (B) (C) by 22 and 15 percent, respectively. The decrease of TRP and increase of KYN metabolites are changes that advance inflammation and indicate cellular immune activation. Changes of both (B & C) were significantly increased over the depletion concentration at (A) (p = 0.03586), followed by a slight, statistically insignificant decrease (0.09509) at 3 months (C); (A-C) remained increased (p<0.0394). Whether the TRP values at (B) (C) (Fig 1A and 1B and Table 1) indicate a new/higher baseline is uncertain. Consistent with the initial decrease in tryptophan at 6–12 hour treatment, all four metabolites in the serotonin/melatonin pathway, including 5-OH-tryptophan and 5-hydroxyindoleacetic acid (5-HIAA; Time A vs B p-value 0.03298 and Time A vs C p-value 0.02551) followed a similar pattern (Fig 2A and 2B).

**Table 1:** Tryptophan/Kynurenine pathway metabolite concentrations in T1D patients.

Patient	Trp	Kyn	KA	AA	XA	PA	QA	3OHAA	mg/dL	mEq/L	mg/mL	Age	Disease	Gender	Race	Diastolic
	uM	nM	nM	nM	nM	nM	nM	Peak Area	Glucose	CO2	BUN	years	Duration			Abnormal
1A	39.4	307.5	862.8	186.3	639.3	1209.0	667.8	9.57E+05	595	<5	26	13.25	1d	M	C	N
1C	66.3	1173.6	1299.8	283.3	711.3	1183.4	724.2	6.31E+05								
2A	41.5	411.1	530.2	294.6	370.3	890.0	510.0	6.14E+05	416	<5	22	14.42	5y	F	AA	Y
2B	51.4	633.6	466.1	128.2	432.4	831.8	618.8	1.52E+05								
2C	45.2	362.8	626.9	172.6	390.3	1087.2	571.8	2.03E+05								
3A	52.7	429.5	530.4	327.4	430.3	1402.4	516.7	8.43E+05	338	5	18	13.58	9y	F	C	Y
3B	94.3	713.6	658.0	279.9	782.3	1349.3	553.5	3.46E+05								
3C	56.3	793.7	461.8	264.1	447.8	1102.2	649.6	2.94E+05								
4A	44.0	737.0	671.3	296.0	386.6	1139.6	450.2	3.33E+05	441	7	20	16.92	3y	M	AA	Y
4B	67.0	1091.5	735.0	266.1	537.2	1697.8	646.7	2.22E+05								
4C	41.8	750.7	788.1	242.8	329.9	1272.0	543.2	2.37E+05								
5A	41.1	203.7	555.0	223.9	351.6	900.5	526.7	9.12E+05	299	5	14	11.42	3y	M	AA	N
5B	56.1	365.6	582.9	271.5	452.7	1316.7	454.0	4.84E+05								
5C	46.7	438.2	506.8	269.3	354.2	811.6	475.2	3.21E+05								
6A	44.3	516.1	986.9	325.4	388.3	983.0	473.1	1.01E+06	845	7	42	10.08	1d	F	C	N
6B	52.3	702.5	626.4	188.9	424.0	926.7	513.4	2.65E+05								
6C	69.9	459.6	889.7	303.9	601.9	1263.3	435.9	5.11E+05								
7A	60.2	454.5	563.2	146.0	564.0	898.6	549.8	4.83E+05	479	7	25	16.92	1y	M	AA	N
7B	50.7	543.0	553.1	286.8	441.9	837.9	645.4	6.38E+05								
7C	45.2	408.9	629.5	229.0	374.5	661.7	511.8	2.44E+05								
8A	28.3	258.9	467.4	177.9	238.8	677.4	476.4	2.06E+05	322	11	15	16.33	1d	F	AA	Y
8B	36.8	315.3	440.8	127.1	316.3	795.7	537.5	2.12E+05								
8C	34.9	313.2	585.2	97.0	296.2	842.3	553.5	1.77E+05								
9A	25.5	295.0	376.0	141.8	210.2	516.9	365.9	2.70E+05	541	7	18	13.08	1d	M	C	N
9B	36.2	291.4	298.7	103.3	286.4	434.1	465.6	1.12E+05								
9C	37.0	226.6	888.6	164.6	361.7	998.3	388.0	6.87E+05								
10A	25.3	379.3	549.1	148.9	249.5	759.2	388.2	5.36E+05	552	<5	15	14.33	12y	F	AA	Y
10B	36.2	280.1	562.5	218.7	271.3	702.6	359.9	2.27E+05								
10C	31.9	682.9	452.2	179.9	250.9	856.7	469.0	2.93E+05								
11A	19.3	185.8	308.0	153.8	207.7	470.6	434.1	2.32E+05	576	6	14	11.58	1d	F	AA	Y
11B	31.0	314.1	413.4	169.4	243.9	969.9	336.9	3.85E+05								
11C	34.7	305.8	401.4	93.3	254.7	829.6	494.0	3.09E+05								
12A	31.2	918.2	815.3	177.4	350.0	1222.4	372.9	5.97E+05	260	7	31	16.25	6y	M	AA	Y
12B	48.4	367.6	445.2	59.6	414.4	1014.0	487.7	5.88E+05								
12C	38.3	356.1	535.1	98.4	338.3	924.5	446.2	7.00E+05								
13B	32.7	365.1	470.1	130.4	274.6	868.2	430.7	4.82E+05	458	<5	12	15.25	7y	F	C	N
13C	34.2	395.8	300.6	158.1	262.8	875.3	379.6	4.10E+05								
14A	36.2	430.8	262.2	105.5	262.2	562.9	449.4	1.15E+05								
14B	29.3	296.8	475.9	203.1	227.2	1057.5	366.4	4.00E+05	550	6	22	9.67	1d	F	AA	N
14C	29.0	258.9	362.1	128.6	233.9	955.8	405.1	2.84E+05								
15A	23.9	412.7	505.4	164.3	237.3	676.1	349.3	4.31E+05	560	6	18	15.5	13y	F	AA	Y
15B	32.1	288.8	404.3	127.8	268.0	745.5	480.4	2.42E+05								
15C	36.3	361.7	448.1	139.4	290.8	631.7	369.2	5.11E+05								

Concentrations of tryptophan/kynurenine pathway metabolites tryptophan (Trp), kynurenine (Kyn), quinolinic acid (QA), kynurenic acid (KA), 3-OH-anthranilic acid (3OHAA), anthranilic acid (AA), picolinic acid (PA), xanthurenic acid (XA) in 15 patients at three time points, Time A at 6-12hours, Time B at 2 weeks and Time C at 3 months after the beginning of treatment. The patient glucose levels, urea nitrogen (BUN), CO_2_ concentration, age, disease duration, gender, race (African American = AA and Caucasian = C) and diastolic abnormality is also listed.

Shan, *et al*., reported that the NMDA gene expression of QA occurs within 4 hours, and possibly as early as 30 minutes [50]. This is in keeping with the 4–12 hour “warning” after treatment is initiated [25, 26] when signs and symptoms are more likely to occur due to increasing intracranial pressure (ICP) that was present pretreatment [27]. It is possible that KYNA’s early modulation of QA still allows QA to be involved, but to a lesser degree, in the pathogenesis of pretreatment subclinical brain [27] and pulmonary edema [29], depending on the degree of amino acid blockade of KYNA [51].

**Fig 3:**
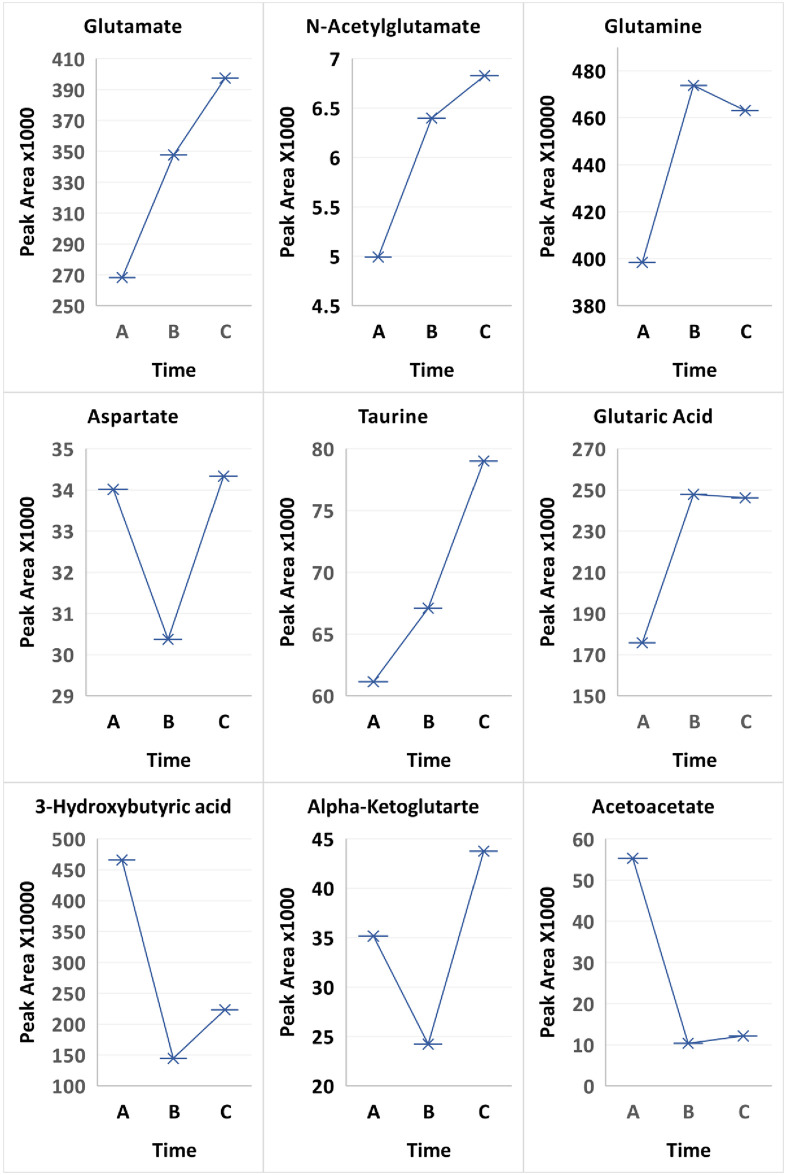
Comparison of treatment over 3 time points Time A at 6-12hours, Time B at 2 weeks and Time C at 3 months between all patients for metabolites glutamate, N-acetylglutamate, glutamine, aspartate, taurine, glutaric acid, acetoacetate and 3-hydroxybutyric acid.

Our study is in keeping with reports of vascular perturbation by TRP/KYN’s ability to modify membrane fluidity [52] and increase albumin permeability in rat brain microvessels [53]. Additional systemic molecules that have been studied longitudinally are likely increased by the suboptimal metabolic/immunologic control during DKA progression prior to treatment. The candidates included as potential mediators/perturbators of CCEC are: 1) VEGF, ET-1, ICAM-1 [47–49]; 2) low background inflammatory molecules especially cytokines prior to the SIR [11, 54, 55]; 3) C3a and C5a complement fragments [14, 56]; and 4) heat shock protein 70-kDa, a modulator of cellular activity/protection and protein homeostasis [57, 58]. This is the first systemic longitudinal report of the excitatory amino acids (EAAs) in uncomplicated severe DKA. This extensively studied system in the brain involves aspartate, glutamine, glutamate [59], n-acetyl-glutamate and taurine, a protective neuronal metabolite [60, 61]. Only aspartate was increased during treatment (A), decreased at two weeks (B), and rebounded at 3 months to greater than (A). At time (A) there was decrease in glutamine and glutamate, with the greatest difference in N-acetylglutamate (p-value 0.0216 for Time A vs. B and 0.015552 for Time A vs. C) and for taurine (p-value 0.0838 for Time A vs. C; Fig 3). The EAAs in the extracellular fluid of vasogenic BE are involved in the pathogenesis of initial and secondary neuronal injuries and inhibited by KYNA modulation of the AA receptors [62, 63]. This too indicates a possible role for EAAs in the pathogenesis of DKA’s pretreatment subclinical BE [27, 29].

Pierozan et al.’s KP studies, unrelated to T1D [64, 65], involve the BBB/brain and suggests TRP/KP metabolites such as QA have the potential to offer insight into the pathogenesis of the BE of DKA. They reported the toxicity of QA disrupts the phosphorylation associated with intermediate filament structural proteins of astrocytes and neurons. An increased duration of QA exposure for 72 hours versus 24 hours, with the longer time containing a 90 percent lower concentration of QA, has a greater toxicity than the higher QA concentration for 24 hours [66]. This supports an extended injury and is important since QA has a number of toxic mechanisms in addition to excitotoxicity and oxidative stress [67]. Another consideration for perturbation is that the IV initial hydration influences a transient further decrease in pH, and a paradoxical increase of the SIR [11, 55], both of which can worsen the initial pathogenesis of the inflammation.

In order to prevent clinical BE or slow progressive molecular perturbation, an earlier time than is presently suggested (4–12 hours) is important. This is supported by the studies of Vavilala, et al., who reported a “decreased transit time” and an increased “apparent diffusion coefficient” between the studies at 12–24 and 36–72 hours, along with an increase in BBB permeability [68, 69]. Parsing the KP metabolites in terms of their early potential systemic toxicity of organ tissue could possibly lead to more certain monitoring of the progressive pathogenesis of BE and the establishment of effective intervention earlier in treatment. This early sequence of subclinical BE takes into consideration the “warning” signs of clinical BE that (a 4–12 hour interval after starting treatment) were published in 2004 [26] prior to the recent reports of the perturbing effects of oxidative [4, 5] and inflammatory [8–16] DKA stresses.

Immunohistochemistry studies of tight junction proteins (TJP) in fatal BE/DKA identified: a) astrocyte hypertrophy and distortion of configuration [70]; and b) BBB disruption due to fragmentation [71]. In addition, other inflammatory markers in the fatal BE/DKA capable of perturbation are: CCL2 and Iba-1 [71]; IL-1 beta [72]; C5b-9 [73]; and MMP-9 [15, 16], with marked cerebral oxidative stress (5). QA’s important early role in the pathogenesis of the BE/DKA, its pretreatment molecular interaction with the AGE-RAGE inflammatory pathway, and the severe metabolic and immunologic dysregulation of DKA were reported in an undiagnosed and untreated young woman with new onset T1D and fatal DKA, who had myocardial RAGE expression at autopsy [10]. This pretreatment RAGE activation is in keeping with the study of Serratos, *et al*. [74] who reported the QA initiation of RAGE expression. Similarly, QA’s brain activation of RAGE is evident in the treated fatal DKA/BE where QA is also likely to have induced astrocyte activation and chemokine production [70, 75]. RAGE expression both in the brain [70] and myocardium [10] supports an early QA interaction possibly involving an incomplete modulation of QA by KYNA [76, 77], and an incomplete suppression of NMDAR by the correction of acidosis [78].

Sequential transcranial Doppler ultrasounds, also provide information of pretreatment autoregulation and vascular hemodynamics [79, 80]. Wootton-Gorges et al. used proton MR with N-acetylaspartate to creatine ratios to identify acute insults in relation to and following DKA treatment. The hypothesis of an early and possibly transient KP inflammatory neuronal insult was reported at the early time of two hours and dissipated at 72 hours [81]. In another study of theirs, one patient with repeated episodes of DKA had markers of persistent insult indicating permanent neuronal damage [82].

Neuroradiologic studies of fatal BE/DKA, that are not sequential, have been reported on frequent occasions in young patients [83, 84]. Without reference to potential time- associated systemic biomarkers of the x-ray damage, it is difficult to evaluate the pathogenesis of the BE of DKA, especially when referring to the “natural history” of a life-threatening metabolic and immunologic crisis [71]. With biomarkers, these studies might possibly contribute to the pathogenesis of DKA/BE. When comparing different etiologies and pathogenesis of clinical BE, one also needs to consider the difference in the dynamics of the acute medical event such as trauma (TBI), where disruption of the BBB occurs in a less toxic milieu and the BE prodromal period is likely to be considerably shorter.

The molecular interactions of DKA raise interesting questions: 1) Does the modulated decrease of QA by KYNA (KA) cause a dampening/blockage of the interaction between QA and NMDA receptor (NMDAR) and thus frequently prevents clinical BE? 2) Does DKA’s acidotic milieu significantly and variably block NMDAR activation [78]. The possibilities in questions 1 and 2 appear to differ from the usually considered hypothesis that the severity of acidosis (on admission) is a part of the milieu that mediates the progression of BE [85, 86], which could still be possible via other toxic mediators even without the KP. 3) Does increased age explain a lower prevalence of DKA/BE in older T1D patients, [87] due to the decreasing selectivity of NMDAR vulnerability with increasing age [87, 88]. The importance of these questions is based on the “unpredictable” KP molecular interactions. An example of this unpredictability is the increase of KYNA, a neuroprotector in the STZ rat, that also has a negative impact on cognition and emotions [89–91].

**Fig 4:**
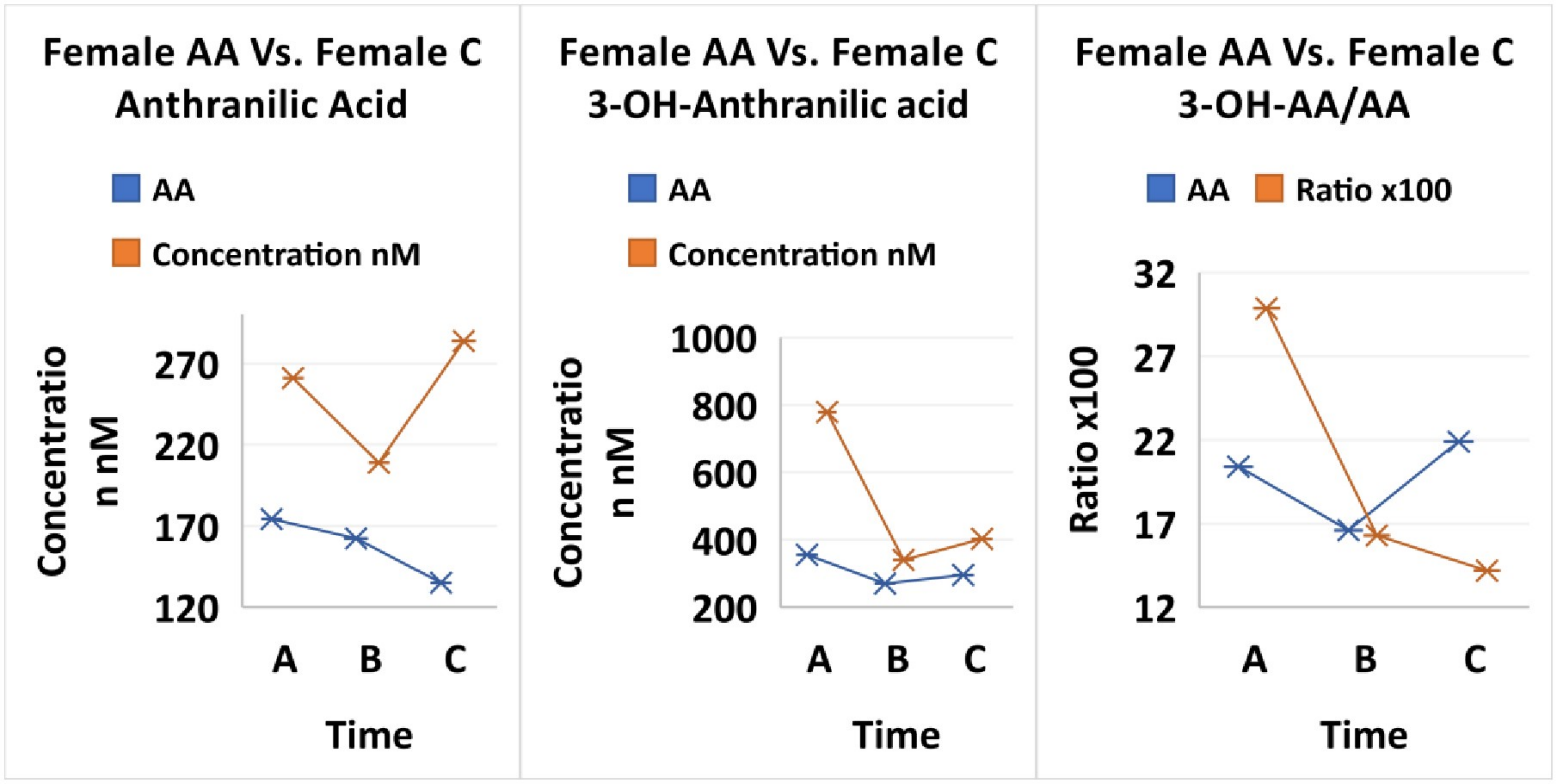
Comparison of treatment over 3 time points Time A at 6-12hours, Time B at 2 weeks and Time C at 3 months between African American females and Caucasian females for 3-OH-antrhranilic acid (3OHAA), anthranilic acid (AA), and 3OHAA/AA.

Interesting observations in our study are seen in the comparisons with KP studies by Darlington, et al. [92]; and Badawy and Dougherty [93] in context of differences between races and gender. The observations involve considering the 3-OHAA/AA ratio, that has biological importance in numerous medical conditions and possibly a racial significance. The ratio importance is in part mediated by the redox active compound 3-OHAA and its strong metabolite interactions. As a marker for inflammation, there is most frequently a systemic decrease in 3-OHAA, and an increase in AA in a range of neurological and other disorders [92]. In our T1D young patient study, the two metabolites throughout treatment are higher in the Caucasian females compared to their African American female peers (Fig 4). While the 3OHAA/AA ratio and 3OHAA in African American females did not change much throughout treatment, Caucasian females experienced a greater initial response to treatment (Fig 9). Badawy’s also saw a lower ratio of 3OHAA/AA and lower 3OHAA in an older and larger group of African American females compared to Caucasian females [93].

Darlington pointed out that a low 3OHAA/AA ratio correlated with stroke victims and decreased survival rate. African Americans with T1D, and their lower 3OHAA/AA, may be at higher risk to stroke and death. In addition, 3OHAA has the ability to depress the release of cytokines from T cells as well as an anti-proliferative effect and suppress the pro-inflammatory transcription Nuclear Factor kappa-B (NF-kB). The lower levels of 3OHAA in African American women with T1D may also put them at higher risk to the deleterious effects of the immune system inflammatory response to viral infections.

**Fig 5:**
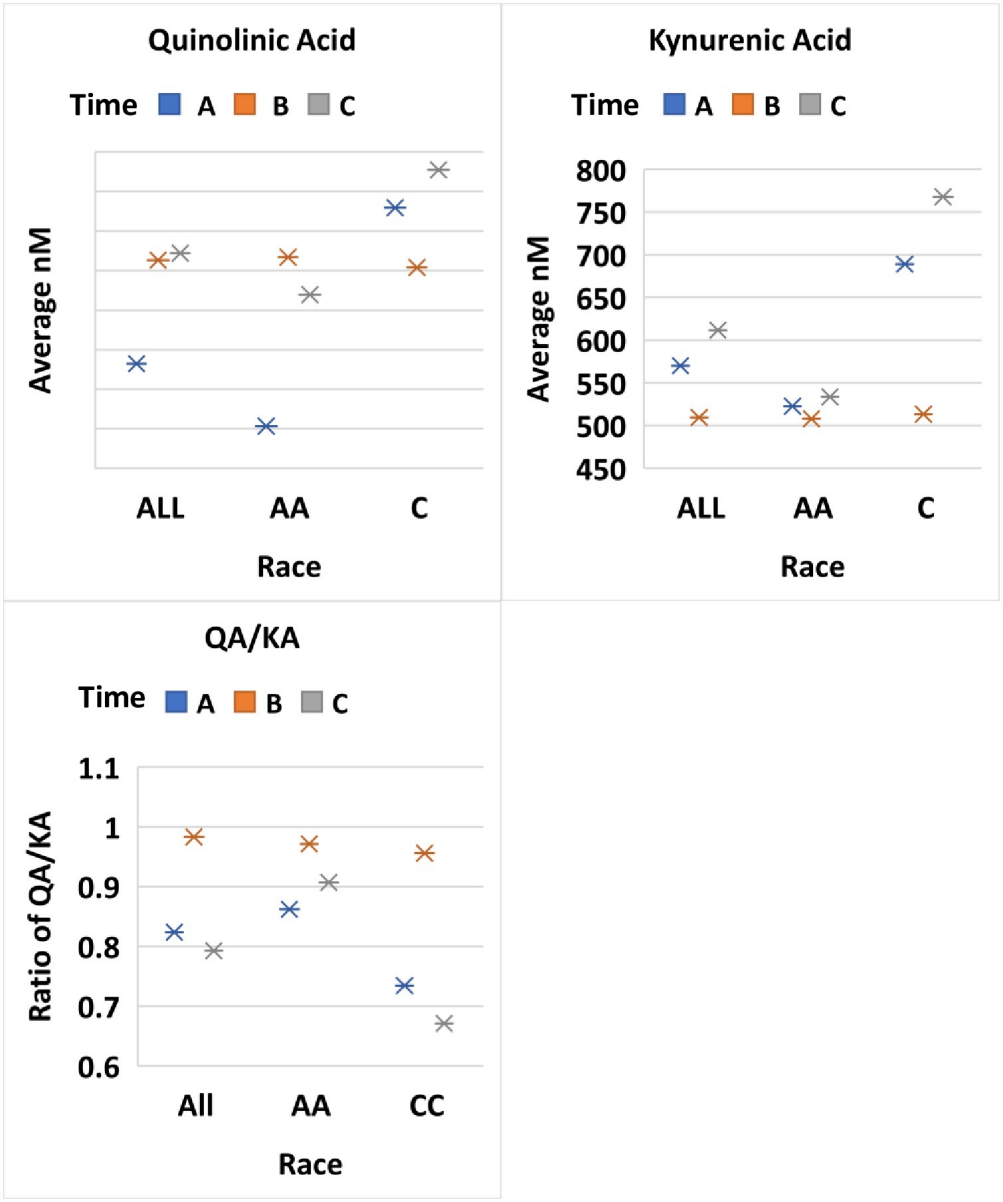
Comparison of treatment over 3 time points Time A at 6-12hours, Time B at 2 weeks and Time C at 3 months between all patients, African Americans and Caucasians for metabolites Quinolinic Acid (QA), Kynurenic Acid (KA) and QA/KA.

**Fig 6:**
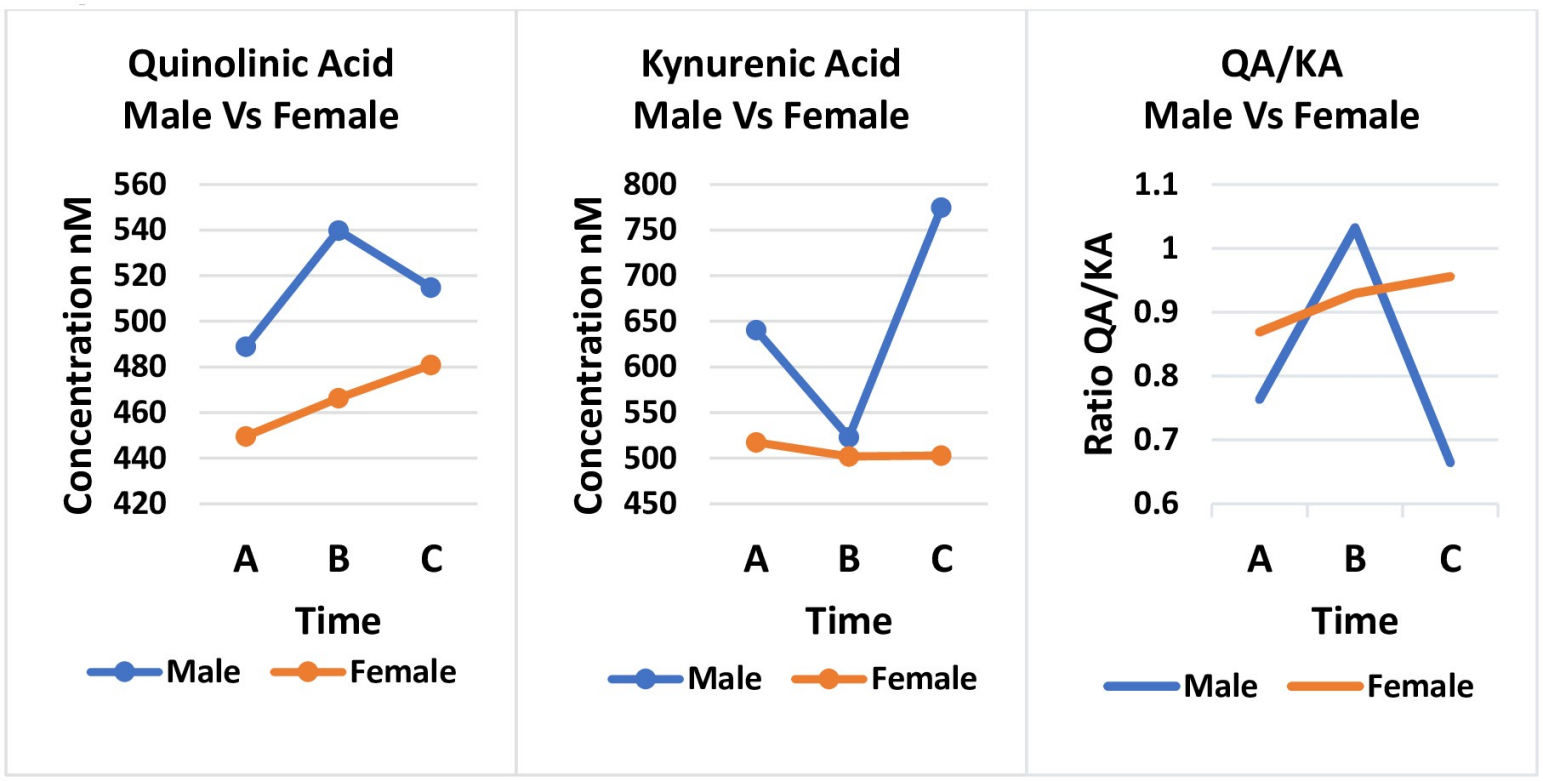
Comparison of treatment over 3 time points Time A at 6-12hours, Time B at 2 weeks and Time C at 3 months between males and females for metabolites Quinolinic Acid (QA), Kynurenic Acid (KA) and QA/KA.

Both 3OHAA and AA are involved at the level of the enzyme 3OHAA oxidase (3HAO) which exists in central neurons. AA is an effective inhibitor of 3HAO reducing the conversion of 3OHAA to QA and picolinic acid. In normal human populations it is believed that endogenous AA will limit QA formation. Therefore, it has been speculated that lower levels of 3OHAA/AA in patients is a compensatory mechanism to reduce QA toxicity. Interestingly, we saw the greatest decrease in QA in the initial treatment of African American females compared to Caucasian females (Fig 5). However, we saw very little change in KA in African American females and the levels were lower than found in Caucasian females, which is opposite reported by Badawy in older normal females. Similar to Badawy, we saw higher concentrations of KA in the male versus females (Fig 6).

Whether the T1D/DKA race or group size influence the ratio to these differences is uncertain. However, also of immunological interest in racial differences, Ness *et al*. [94] reported a significant demographic association between the differential distribution of allelic variants in cytokine genes; specifically, that African American females are significantly more likely than Caucasian females to carry an allelic variant that upregulates proinflammatory cytokines and down-regulates the anti-inflammatory cytokine IL-10.

**Fig 7:**
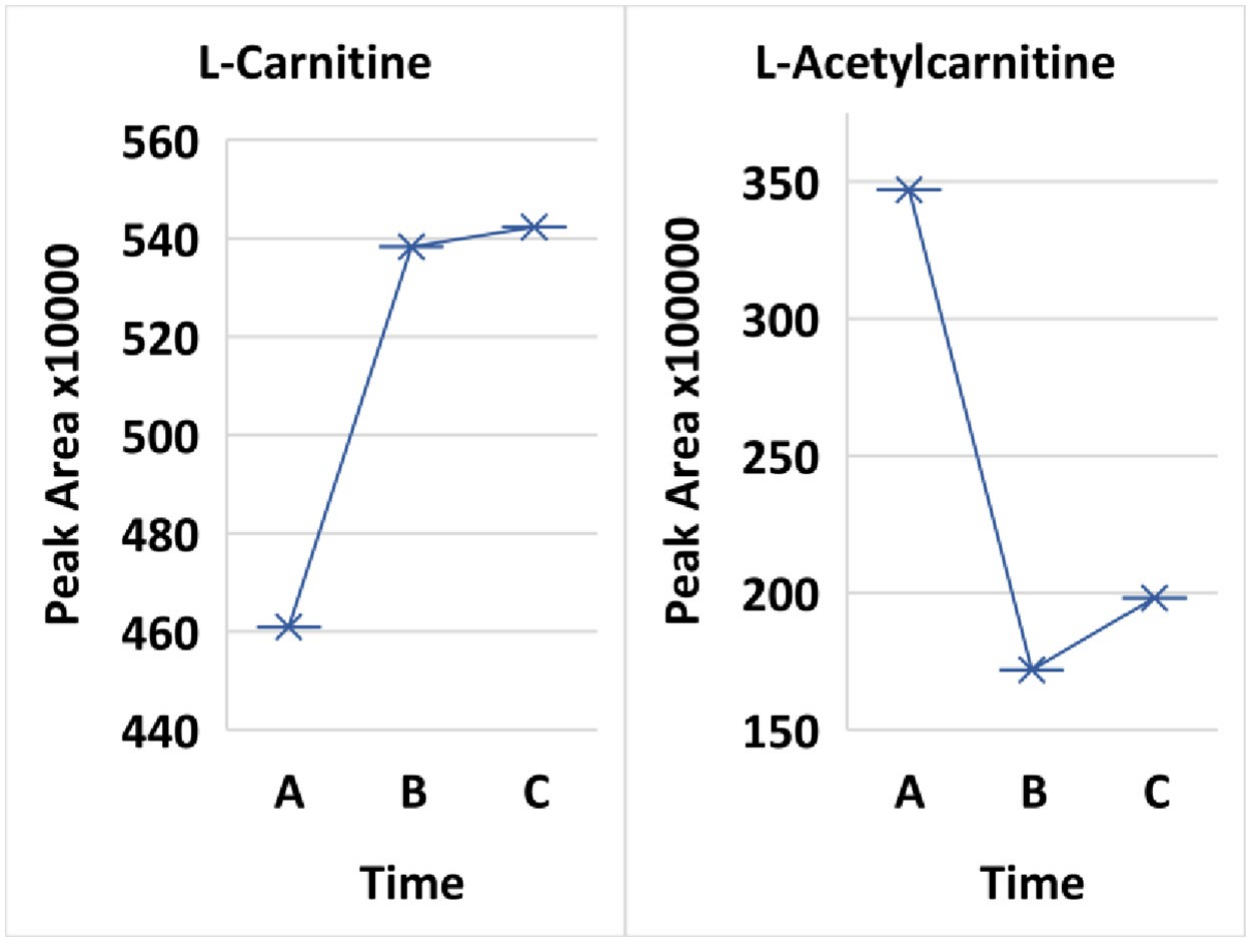
Comparison of treatment over 3 time points Time A at 6-12hours, Time B at 2 weeks and Time C at 3 months for L-carnitine and L-acetylcarnitine.

Diabetic ketoacidosis may be monitored by the ketones 3-hydroxybutaric acid and acetoacetate in the urine. Interestingly, sustained treatment of the patients resulted in significant decreases in the ketones 3-hydroxybutaric acid (p-value 0.0118 Time A versus Time B) and acetoacetate (p-value 0.00326 for Time A versus Time B). Hyperglycemia results in increased lipolysis and a switch in the liver to ketone body formation via fatty acid oxidation. In fatty acid oxidation, carnitine and L-acetylcarnitine cycle (in the reaction acetyl-CoA + carnitine ⇌ CoA + acetylcarnitine) to bring energy to the mitochondria. L-acetylcarnitine was significantly higher at the 6–12 hour treatment than at 2 weeks or 3 months (p-value 0.00066 and 0.002636, respectively) while carnitine levels inversely correlated (Fig 7).

**Fig 8:**
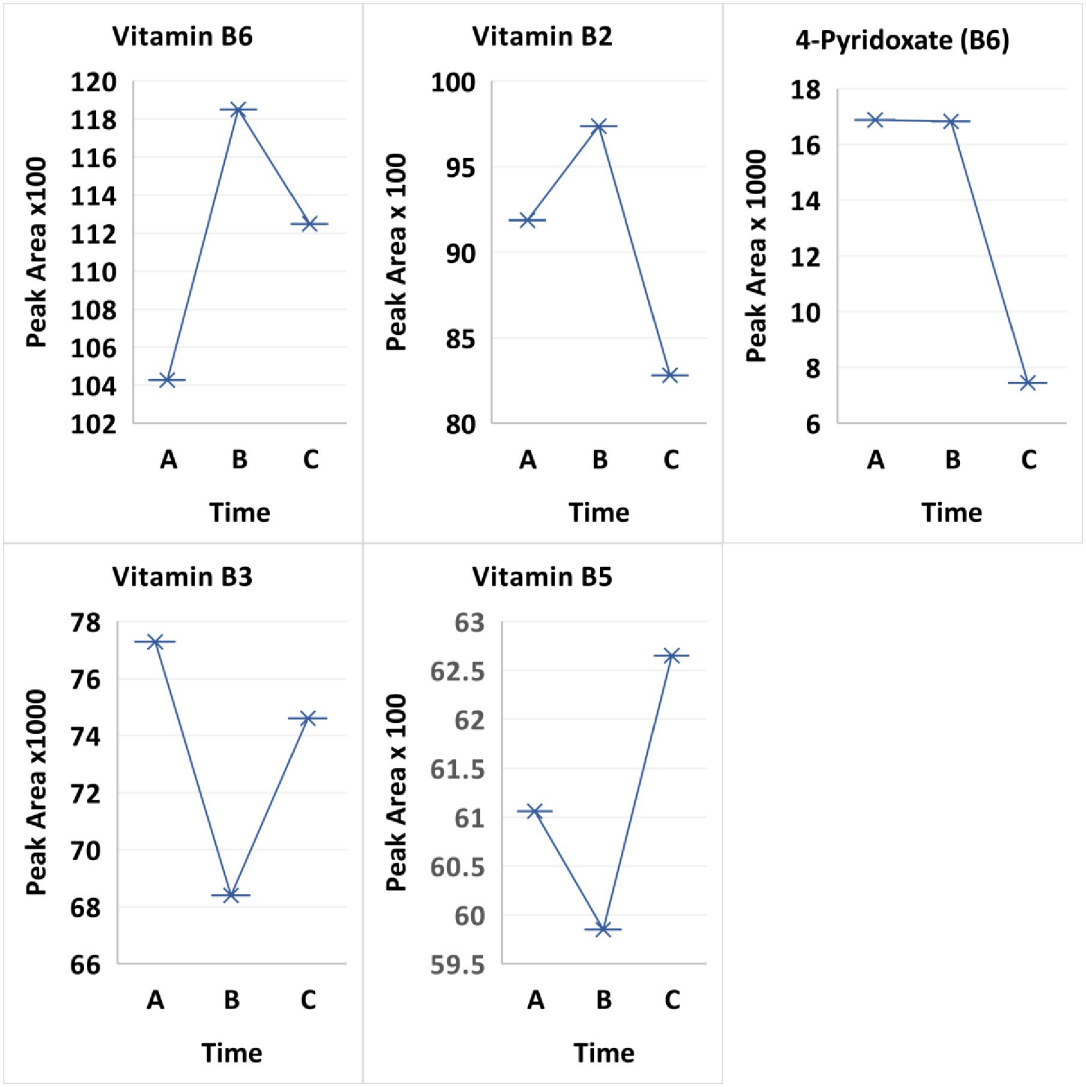
Comparison of treatment over 3 time points Time A at 6-12hours, Time B at 2 weeks and Time C at 3 months between all patients for metabolites vitamin B6, 4-pyridoxate the catabolic product of vitamin B6 and vitamin B2.

Vitamin metabolism has received limited investigation of the water-soluble vitamins in poorly controlled T1D. The vitamins B2 and B6 (pyridoxine) are cofactors in the regulation of basic cellular metabolism during, DKA and their involvement in: 1) endothelial dysfunction, especially in young patients with T1D, [95]; and 2) cardiomyopathy in T1D [96] are markers of the substrate product ratios in the KP (Ulvik, [97]. We studied B2 and B6 at the three time points. B6 had a dramatic compensatory rise at 2–3 weeks (B) after a nadir during treatment (A). At 3 months (C), the B6 concentration had decreased by one-third of the maximum value at (B). B2, riboflavin, rose from the treatment value (A) to the maximum value at 2–3 weeks (B), possibly through the “salvage pathway”, and then declined to its lowest concentration at 3 months (C). Interestingly, the Vitamin B6 catabolic metabolite typically measured in urine, 4-pyridoxic acid, increased in plasma significantly at treatment time A (p-value 0.011002) and B (p-value 0.012504) compared to 3 months (Fig 8).

An important comparison is the strides made in psychology and psychiatry, disciplines associated with diabetes mellitus. However, minimal attention has been directed to KP as a source of perturbation during DKA in individuals with T1D. Unfortunately, we have no information about the KP impact of T1D in this cohort relative to neurocognition and mental health. In a study of young patients with DKA/T1D Jessup *et al*. [31] reported significant, selective, delayed neurocognition that was not clinically apparent after uneventful correction of severe DKA. Similarly, based on low verbal IQs, sustained insults of cognition and learning were reported by Semenkovich *et al*. [98]. The deficits were mediated by poor glycemic control in a young group similar to ours with T1D. An important observation from our DKA study is that KP has a gradual increase of QA concentrations between 2–3 weeks (B) and 3 months (C). This increase of KP is likely to be involved in the pathogenesis of the posttraumatic stress symptoms such as anxiety/depression [99–102] that are experienced by children after being diagnosed with T1D, and also result in structural subclinical brain injury [103].

**Fig 9:**
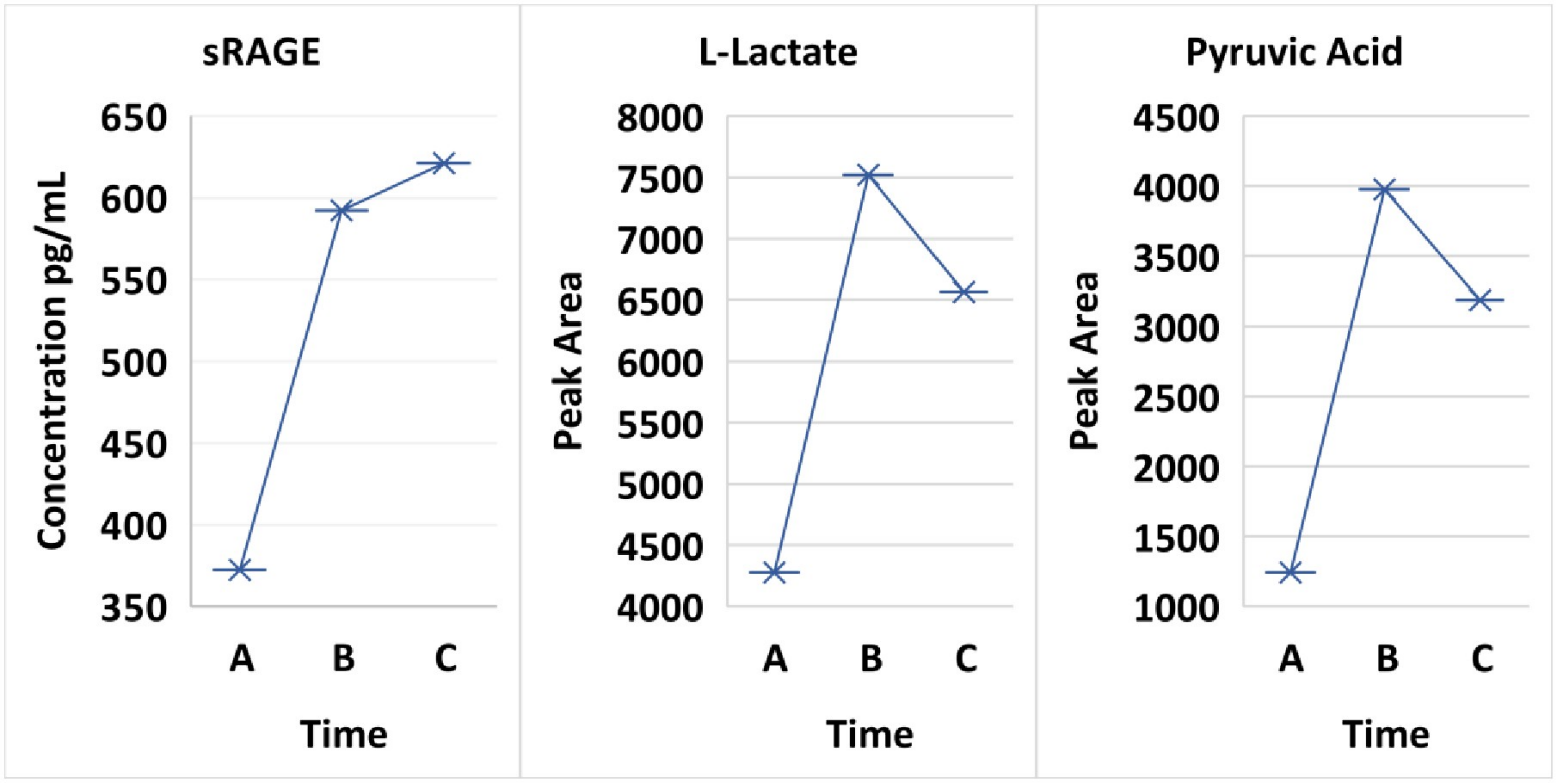
Comparison of sRAGE to L-lactate and pyruvic acid.

Finally, the continued unfortunate prevalence of sudden cardiac death in young people with diabetes remains a major challenge [104]. KYN an inflammatory sensor, is a modulator and a systemic biomarker of congestive heart failure [105]. A low systemic level of TRP is a biomarker of cardiovascular inflammation [105], and an increase of plasma TRP is associated with a decrease of CVD [106]. Colop, *et al*., [107] have reviewed the involvement of the KP in stroke of T1D. These KP contributions to the pathogenesis of CVD are important especially when considering the advances made by the research involving the inflammatory pathway of the AGE-RAGE axis [108]. The recognized ubiquity and variability of the KP in the pathogenesis of acute and chronic illnesses are prevalent; yet the inflammatory role of the KP metabolites and their impact on cardiovascular disease has progressed slowly. Important opportunities for TRP/KP and T1D research are likely based on: 1) the presence of KP ligands in non-diabetic cardiovascular tissues [109]; 2a) the expression of systemic inflammatory cytokines in the myocardium of fatal DKA [110]; and 2b) de-novo synthesis of myocardial autoantibodies in young T1D patients during the correction of uncomplicated DKA [32]; and 3) our present data is supportive for the pathogenesis of KP in cardiac dysfunction in that the systemic concentrations of QA and sRAGE interact in uncomplicated DKA [74] early in treatment (A) and subsequently increase at baseline following correction (B & C) (Fig 9). sRAGE (Time A vs Time B p-value 0.05805) followed a consistent decrease pattern in the initial treatment with L-lactate, pyruvic acid (Time A vs B p-value 0.05155 and Time A vs C p-value 0.02437), KYN and QA. The foundation for the increased awareness of contributions to the pathogenesis fostered by the KP in DKA is an increasing recognition of their relationship between metabolism, oxidative stress and immune inflammation.

## Conclusion

We extend the observation that DKA’s metabolic and immune inflammation are significantly interrelated with the KP as an important perturbation component likely prior to, during and after DKA correction. A neurotoxic candidate for initiating BE, such as QA, is formed early in treatment prior to the warning signs of progressive subclinical BE and is possibly also involved in the early pathogenesis of acute microvascular insults and chronic DKA complications. The inflammatory KP pathway adds molecular intermeshing between its own molecules, in addition to interactions with inflammatory and non-inflammatory molecules of other pathways. We have shown that the KP perturbation and these interactions differ between young AAs and Caucasians, and possibly other races.

As has been suggested in both acute and chronic medical conditions: 1) the increasing inflammatory background of T1D prior to DKA initiates the early transient TRP degradation and the early multifunctional KYN pathways; 2) IDO activation is initiated by inflammatory cytokines and possibly by BOHB [45]; 3) molecular interactions and modulations suggest excitotoxicity does occur in DKA, but whether it occurs via NMDA or another mechanism is uncertain; 4) the study also suggests that the dysregulated milieu has unrecognized molecules some of which are potential perturbators; and 5) KP’s early inflammatory insult during severe DKA requires further investigation of pretreatment and early biomarkers. Finally, the demonstration of the KP in the inflammatory insult of DKA gives the opportunity to refocus on this medical crisis and suggests consideration of modifications in treatment.

The limitations of our study are the: 1] limited number of patients; 2] limited number of TRP/KP sampling times; 3] infrequent sequential systemic BOHB measurements; 4] absence of a nutritional history for the 12–24 hours prior to admission and prior to the (B & C) samples; and 5] lack of sequential neurocognitive testing at (B & C) post DKA correction.

## Supporting information

S1 Data (XLSX)

S2 Data (XLSX)

## References

[1] Durr JA, Hoffman WH, Hensen J, Sklar AH, El Gammal TE, Steinhart CM. Osmoregulation of vasopressin in diabetic ketoacidosis. Am J Physiol—Endocrinol Metab. 1990;259. doi: 10.1152/ajpendo.1990.259.5.E723 2122740

[2] Deeter KH, Roberts JS, Bradford H, Richards T, Shaw D, Marro K, et al. Hypertension despite dehydration during severe pediatric diabetic ketoacidosis. Pediatr Diabetes. 2011;12: 295–301. doi: 10.1111/j.1399-5448.2010.00695.x 21443581 PMC3103609

[3] Hoffman WH, Passmore GG, Hannon DW, Talor M V., Fox P, Brailer C, et al. Increased Systemic Th17 Cytokines Are Associated with Diastolic Dysfunction in Children and Adolescents with Diabetic Ketoacidosis. PLoS One. 2013;8. doi: 10.1371/journal.pone.0071905 24013901 PMC3754936

[4] Lee DM, Hoffman WH, Carl GF, Khichi M, Cornwell PE. Lipid peroxidation and antioxidant vitamins prior to, during, and after correction of diabetic ketoacidosis. J Diabetes Complications. 2002;16: 294–300. doi: 10.1016/s1056-8727(01)00215-x 12126788

[5] Hoffman WH, Siedlak SL, Wang Y, Castellani RJ, Smith MA. Oxidative damage is present in the fatal brain edema of diabetic ketoacidosis. Brain Res. 2011;1369: 194–202. doi: 10.1016/j.brainres.2010.10.085 21040714 PMC3056460

[6] Liebich HM. Gas chromatographic profiling of ketone bodies and organic acids in diabetes. J Chromatogr. 1986;379: 347–66. doi: 10.1016/s0378-4347(00)80689-1 3090087

[7] Katsuki H, Okuda S. Arachidonic acid as a neurotoxic and neurotrophic substance. Prog Neurobiol. 1995;46: 607–636. doi: 10.1016/0301-0082(95)00016-o 8545546

[8] Hoffman WH, Kappler F, Passmore GG, Mehta R. Diabetic ketoacidosis and its treatment increase plasma 3-deoxyglucosone. Clin Biochem. 2003;36: 269–273. doi: 10.1016/s0009-9120(03)00030-4 12810155

[9] Allaman I, Bélanger M, Magistretti PJ. Methylglyoxal, the dark side of glycolysis. Front Neurosci. 2015;9: 1–12. 25709564 10.3389/fnins.2015.00023 PMC4321437

[10] Hoffman WH, Ishikawa T, Blum J, Tani N, Ikeda T, Artlett CM. Soluble receptor for glycation end-products concentration increases following the treatment of severe diabetic ketoacidosis. JCRPE J Clin Res Pediatr Endocrinol. 2020;12: 160–167. doi: 10.4274/jcrpe.galenos.2019.2019.0076 31514489 PMC7291407

[11] Hoffman WH, Burek CL, Waller JL, Fisher LE, Khichi M, Mellick LB. Cytokine response to diabetic ketoacidosis and its treatment. Clin Immunol. 2003;108: 175–181. doi: 10.1016/s1521-6616(03)00144-x 14499240

[12] Carl GF, Hoffman WH, Blankenship PR, Litaker MS, Hoffman MG, Mabe PA. Diabetic ketoacidosis depletes plasma tryptophan. Endocr Res. 2002;28: 91–102. doi: 10.1081/erc-120004541 12108794

[13] Szabó A, Kenesei E, Körner A, Miltényi M, Szücs L, Nagy I. Changes in plasma and urinary amino acid levels during diabetic ketoacidosis in children. Diabetes Res Clin Pract. 1991;12: 91–97. doi: 10.1016/0168-8227(91)90085-r 1908767

[14] Jerath RS, Burek CL, Hoffman WH, Passmore GG. Complement activation in diabetic ketoacidosis and its treatment. Clin Immunol. 2005;116: 11–17. doi: 10.1016/j.clim.2005.03.004 15925827

[15] Omatsu T, Cepinskas G, Clarson C, Patterson EK, Alharfi IM, Summers K, et al. CXCL1/CXCL8 (GROα/IL-8) in human diabetic ketoacidosis plasma facilitates leukocyte recruitment to cerebrovascular endothelium in vitro. Am J Physiol Endocrinol Metab. 2014;306: E1077–84. doi: 10.1152/ajpendo.00659.2013 24619879

[16] Hoffman WH, Cudrici CD, Boodhoo D, Tatomir A, Rus V, Rus H. Intracerebral matrix metalloproteinase 9 in fatal diabetic ketoacidosis. Exp Mol Pathol. 2019;108: 97–104. doi: 10.1016/j.yexmp.2019.04.008 30986397 PMC6563901

[17] Carl GF, Hoffman WH, Passmore GG, Truemper EJ, Lightsey AL, Cornwell PE, et al. Diabetic Ketoacidosis Promotes a Prothrombotic State. Endocr Res. 2003;29: 73–82. doi: 10.1081/erc-120018678 12665320

[18] Amara U, Flierl MA, Rittirsch D, Klos A, Chen H, Acker B, et al. Molecular intercommunication between the complement and coagulation systems. J Immunol. 2010;185: 5628–5636. doi: 10.4049/jimmunol.0903678 20870944 PMC3123139

[19] Royer LO, Knudsen FS, de Oliveira MA, Tavares MFM, Bechara EJH. Peroxynitrite-initiated oxidation of acetoacetate and 2-methylacetoacetate esters by oxygen: potential sources of reactive intermediates in keto acidoses. Chem Res Toxicol. 2004;17: 1725–1732. doi: 10.1021/tx049821y 15606150

[20] Imahori D, Matsumoto T, Kojima N, Kasei T, Sumii M, Sumida T et al. C. No Title. Chem Pharm Bull. 2018;66: 363–367. 10.1248/cpb.c17-00809 29607901

[21] Bolton SJ, Perry VH. Differential blood-brain barrier breakdown and leucocyte recruitment following excitotoxic lesions in juvenile and adult rats. Exp Neurol. 1998;154: 231–240. doi: 10.1006/exnr.1998.6927 9875284

[22] Stamatovic SM, Keep RF, Andjelkovic A V. Brain endothelial cell-cell junctions: how to “open” the blood brain barrier. Curr Neuropharmacol. 2008;6: 179–192. doi: 10.2174/157015908785777210 19506719 PMC2687937

[23] Mándi Y, Vécsei L. The kynurenine system and immunoregulation. J Neural Transm. 2012;119: 197–209. doi: 10.1007/s00702-011-0681-y 21744051

[24] Savitz J. The kynurenine pathway: a finger in every pie. Mol Psychiatry. 2020;25: 131–147. doi: 10.1038/s41380-019-0414-4 30980044 PMC6790159

[25] Carlotti APCP, Bohn D, Halperin ML. Importance of timing of risk factors for cerebral oedema during therapy for diabetic ketoacidosis. Arch Dis Child. 2003;88: 170–173. doi: 10.1136/adc.88.2.170 12538330 PMC1719453

[26] Dunger DB, Sperling MA, Acerini CL, Bohn DJ, Daneman D, Danne TPA, et al. ESPE/LWPES consensus statement on diabetic ketoacidosis in children and adolescents. Arch Dis Child. 2004;89: 188–194. doi: 10.1136/adc.2003.044875 14736641 PMC1719805

[27] Hoffman WH, Steinhart CM, el Gammal T, Steele S, Cuadrado AR, Morse PK. Cranial CT in children and adolescents with diabetic ketoacidosis. AJNR Am J Neuroradiol. 1988;9: 733–739. 3135717 PMC8332012

[28] Glaser NS, Wootton-Gorges SL, Buonocore MH, Marcin JP, Rewers A, Strain J, et al. Frequency of sub-clinical cerebral edema in children with diabetic ketoacidosis. Pediatr Diabetes. 2006;7: 75–80. doi: 10.1111/j.1399-543X.2006.00156.x 16629712

[29] Hoffman WH, Locksmith JP, Burton EM, Hobbs E, Passmore GG, Pearson-Shaver AL, et al. Interstitial pulmonary edema in children and adolescents with diabetic ketoacidosis. J Diabetes Complications. 1998;12: 314–320. doi: 10.1016/s1056-8727(98)00012-9 9877465

[30] Ghetti S, Lee JK, Sims CE, DeMaster DM, Glaser NS. Diabetic Ketoacidosis and Memory Dysfunction in Children with Type 1 Diabetes. J Pediatr. 2010;156: 109–114. doi: 10.1016/j.jpeds.2009.07.054 19833353

[31] Jessup AB, Grimley MB, Meyer E, Passmore GP, Belger A, Hoffman WH, et al. Effects of Diabetic Ketoacidosis on Visual and Verbal Neurocognitive Function in Young Patients Presenting with New-Onset Type 1 Diabetes. J Clin Res Pediatr Endocrinol. 2015;7: 203–210. doi: 10.4274/jcrpe.2158 26831554 PMC4677555

[32] Hoffman WH, Sharma M, Cihakova D, Talor MV, Rose NR, Mohanakumar T, et al. Cardiac antibody production to self-antigens in children and adolescents during and following the correction of severe diabetic ketoacidosis. Autoimmunity. 2016;49: 188–196. doi: 10.3109/08916934.2015.1134509 26911924

[33] Reyes-Ocampo J, Ramírez-Ortega D, Vázquez Cervantes GI, Pineda B, Montes de Oca Balderas P, González-Esquivel D, et al. Mitochondrial dysfunction related to cell damage induced by 3-hydroxykynurenine and 3-hydroxyanthranilic acid: Non-dependent-effect of early reactive oxygen species production. Neurotoxicology. 2015;50: 81–91. doi: 10.1016/j.neuro.2015.08.003 26254737

[34] Brooks AK, Lawson MA, Smith RA, Janda TM, Kelley KW, McCusker RH. Interactions between inflammatory mediators and corticosteroids regulate transcription of genes within the Kynurenine Pathway in the mouse hippocampus. J Neuroinflammation. 2016;13: 98. doi: 10.1186/s12974-016-0563-1 27142940 PMC4855471

[35] Sperling MA. Fluid Composition, Infusion Rate, and Brain Injury in Diabetic Ketoacidosis. N Engl J Med. 2018;378: 2336–2338. doi: 10.1056/NEJMe1806017 29897856

[36] Liu J-J, Movassat J, Portha B. Emerging role for kynurenines in metabolic pathologies. Curr Opin Clin Nutr Metab Care. 2019;22. Available: https://journals.lww.com/co-clinicalnutrition/Fulltext/2019/01000/Emerging_role_for_kynurenines_in_metabolic.15.aspx doi: 10.1097/MCO.0000000000000529 30407222

[37] Thomas SR, Stocker R. Redox reactions related to indoleamine 2,3-dioxygenase and tryptophan metabolism along the kynurenine pathway. Redox Rep. 1999;4: 199–220. doi: 10.1179/135100099101534927 10731095

[38] Lugo-Huitrón R, Ugalde Muñiz P, Pineda B, Pedraza-Chaverrí J, Ríos C, Pérez-De La Cruz V. Quinolinic acid: An endogenous neurotoxin with multiple targets. Oxid Med Cell Longev. 2013;2013. doi: 10.1155/2013/104024 24089628 PMC3780648

[39] Guillemin GJ. Quinolinic acid, the inescapable neurotoxin. FEBS J. 2012;279: 1356–1365. doi: 10.1111/j.1742-4658.2012.08485.x 22248144

[40] Guillemin GJ, Smythe G, Takikawa O, Brew BJ. Expression of indoleamine 2,3-dioxygenase and production of quinolinic acid by human microglia, astrocytes, and neurons. Glia. 2005;49: 15–23. doi: 10.1002/glia.20090 15390107

[41] Horrocks LA, Farooqui AA. NMDA Receptor-Stimulated Release of Arachidonic Acid: Mechanisms for the Bazan Effect BT—Cell Signal Transduction, Second Messengers, and Protein Phosphorylation in Health and Disease. In: Municio AM, Miras-Portugal MT, editors. Boston, MA: Springer US; 1994. pp. 113–128.

[42] Ganong AH, Cotman CW. Kynurenic acid and quinolinic acid act at N-methyl-D-aspartate receptors in the rat hippocampus. J Pharmacol Exp Ther. 1986;236: 293 LP–299. Available: http://jpet.aspetjournals.org/content/236/1/293.abstract 2867215

[43] Tasker RC, Acerini CL. Cerebral edema in children with diabetic ketoacidosis: Vasogenic rather than cellular? Pediatr Diabetes. 2014;15: 261–270. doi: 10.1111/pedi.12153 24866062

[44] Fiordalisi I, Novotny WE, Holbert D, Finberg L, Harris GD. An 18-yr prospective study of pediatric diabetic ketoacidosis: an approach to minimizing the risk of brain herniation during treatment. Pediatr Diabetes. 2007;8: 142–149. doi: 10.1111/j.1399-5448.2007.00253.x 17550424

[45] Chmiel-Perzyńska I, Kloc R, Perzyński A, Rudzki S, Urbańska EM. Novel aspect of ketone action: β-Hydroxybutyrate increases brain synthesis of kynurenic acid in vitro. Neurotoxicity Research. 2011. pp. 40–50. doi: 10.1007/s12640-010-9220-0 20838951

[46] Yang H, Shan W, Zhu F, Wu J, Wang Q. Ketone bodies in neurological diseases: Focus on neuroprotection and underlying mechanisms. Front Neurol. 2019;10: 1–11. 31244753 10.3389/fneur.2019.00585 PMC6581710

[47] Isales CM, Min L, Hoffman WH. Acetoacetate and β-hydroxybutyrate differentially regulate endothelin-1 and vascular endothelial growth factor in mouse brain microvascular endothelial cells. J Diabetes Complications. 1999;13: 91–97. doi: 10.1016/s1056-8727(99)00030-6 10432173

[48] Hoffman WH, Cheng C, Passmore GG, Carroll JE, Hess D. Acetoacetate increases expression of intercellular adhesion molecule-1 (ICAM-1) in human brain microvascular endothelial cells. Neurosci Lett. 2002;334: 71–74. doi: 10.1016/s0304-3940(02)00816-9 12435474

[49] Jain SK, Kannan K, Lim G, Matthews-Greer J, McVie R, Bocchini JAJ. Elevated blood interleukin-6 levels in hyperketonemic type 1 diabetic patients and secretion by acetoacetate-treated cultured U937 monocytes. Diabetes Care. 2003;26: 2139–2143. doi: 10.2337/diacare.26.7.2139 12832326

[50] Shan Y, Carlock LR, Walker PD. NMDA receptor overstimulation triggers a prolonged wave of immediate early gene expression: Relationship to excitotoxicity. Exp Neurol. 1997;144: 406–415. doi: 10.1006/exnr.1997.6427 9168840

[51] Sekine A, Okamoto M, Kanatani Y, Sano M, Shibata K, Fukuwatari T. Amino acids inhibit kynurenic acid formation via suppression of kynurenine uptake or kynurenic acid synthesis in rat brain in vitro. Springerplus. 2015;4: 48. doi: 10.1186/s40064-015-0826-9 25674503 PMC4318830

[52] Rudzite V, Jurika E, Jirgensons J. Changes in membrane fluidity induced by tryptophan and its metabolites. Adv Exp Med Biol. 1999;467: 353–367. doi: 10.1007/978-1-4615-4709-9_46 10721077

[53] Št’astný F, Škultétyová I, Pliss L, Ježová D. Quinolinic acid enhances permeability of rat brain microvessels to plasma albumin. Brain Res Bull. 2000;53: 415–420. doi: 10.1016/s0361-9230(00)00368-3 11136997

[54] Heier M, Margeirsdottir HD, Brunborg C, Hanssen KF, Dahl-Jørgensen K, Seljeflot I. Inflammation in childhood type 1 diabetes; influence of glycemic control. Atherosclerosis. 2015;238: 33–37. doi: 10.1016/j.atherosclerosis.2014.11.018 25437887

[55] Karavanaki K, Karanika E, Georga S, Bartzeliotou A, Tsouvalas M, Konstantopoulos I, et al. Cytokine response to diabetic ketoacidosis (DKA) in children with type 1 diabetes (T1DM). Endocr J. 2011;58: 1045–1053. doi: 10.1507/endocrj.ej11-0024 22033476

[56] Schraufstatter IU, Trieu K, Sikora L, Sriramarao P, DiScipio R. Complement c3a and c5a induce different signal transduction cascades in endothelial cells. J Immunol. 2002;169: 2102–2110. doi: 10.4049/jimmunol.169.4.2102 12165538

[57] Brown IR. Induction of heat shock (stress) genes in the mammalian brain by hyperthermia and other traumatic events: a current perspective. J Neurosci Res. 1990;27: 247–255. doi: 10.1002/jnr.490270302 2097376

[58] Oglesbee MJ, Herdman AV, Passmore GG, Hoffman WH. Diabetic ketoacidosis increases extracellular levels of the major inducible 70-kDa heat shock protein. Clin Biochem. 2005;38: 900–904. doi: 10.1016/j.clinbiochem.2005.05.011 16009359

[59] Hansson E, Johansson BB, Westergren I, Rönnbäck L. Glutamate-induced swelling of single astroglial cells in primary culture. Neuroscience. 1994;63: 1057–1066. doi: 10.1016/0306-4522(94)90572-x 7535392

[60] Westergren I, Nyström B, Hamberger A, Johansson BB. Amino acids in extracellular fluid in vasogenic brain edema. Acta Neurochir Suppl (Wien). 1994;60: 124–127. doi: 10.1007/978-3-7091-9334-1_33 7976523

[61] Su Y, Fan W, Ma Z, Wen X, Wang W, Wu Q, et al. Taurine improves functional and histological outcomes and reduces inflammation in traumatic brain injury. Neuroscience. 2014;266: 56–65. doi: 10.1016/j.neuroscience.2014.02.006 24530657

[62] Borgens R Ben, Liu-Snyder P. Understanding secondary injury. Q Rev Biol. 2012;87: 89–127. doi: 10.1086/665457 22696939

[63] Swartz KJ, During MJ, Freese A, Beal MF. Cerebral synthesis and release of kynurenic acid: an endogenous antagonist of excitatory amino acid receptors. J Neurosci. 1990;10: 2965–2973. doi: 10.1523/JNEUROSCI.10-09-02965.1990 2168940 PMC6570241

[64] Pierozan P, Ferreira F, de Lima BO, Pessoa-Pureur R. Quinolinic acid induces disrupts cytoskeletal homeostasis in striatal neurons. Protective role of astrocyte-neuron interaction. J Neurosci Res. 2015;93: 268–284. doi: 10.1002/jnr.23494 25306914

[65] Pierozan P, Pessoa-Pureur R. Cytoskeleton as a Target of Quinolinic Acid Neurotoxicity: Insight from Animal Models. Mol Neurobiol. 2018;55: 4362–4372. doi: 10.1007/s12035-017-0654-8 28647871

[66] Chiarugi A, Meli E, Moroni F. Similarities and differences in the neuronal death processes activated by 3OH-kynurenine and quinolinic acid. J Neurochem. 2001;77: 1310–1318. doi: 10.1046/j.1471-4159.2001.00335.x 11389182

[67] Pérez-De La Cruz V, Carrillo-Mora P, Santamaría A. Quinolinic Acid, an endogenous molecule combining excitotoxicity, oxidative stress and other toxic mechanisms. Int J Tryptophan Res. 2012;5: 1–8. doi: 10.4137/IJTR.S8158 22408367 PMC3296489

[68] Vavilala MS, Marro KI, Richards TL, Roberts JS, Curry P, Pihoker C, et al. Change in mean transit time, apparent diffusion coefficient, and cerebral blood volume during pediatric diabetic ketoacidosis treatment. Pediatr Crit care Med a J Soc Crit Care Med World Fed Pediatr Intensive Crit Care Soc. 2011;12: e344–9. doi: 10.1097/PCC.0b013e3182196c9c 21516055 PMC3157541

[69] Vavilala MS, Richards TL, Roberts JS, Chiu H, Pihoker C, Bradford H, et al. Change in blood-brain barrier permeability during pediatric diabetic ketoacidosis treatment. Pediatr Crit care Med a J Soc Crit Care Med World Fed Pediatr Intensive Crit Care Soc. 2010;11: 332–338. 19838141 10.1097/PCC.0b013e3181c013f4 PMC2913885

[70] Hoffman WH, Artlett CM, Zhang W, Kreipke CW, Passmore GG, Rafols JA, et al. Receptor for advanced glycation end products and neuronal deficit in the fatal brain edema of diabetic ketoacidosis. Brain Res. 2008;1238: 154–162. doi: 10.1016/j.brainres.2008.08.041 18775683

[71] Hoffman WH, Stamatovic SM, Andjelkovic AV. Inflammatory mediators and blood brain barrier disruption in fatal brain edema of diabetic ketoacidosis. Brain Res. 2009;1254: 138–148. doi: 10.1016/j.brainres.2008.11.100 19103180

[72] Hoffman WH, Casanova MF, Cudrici CD, Zakranskaia E, Venugopalan R, Nag S, et al. Neuroinflammatory response of the choroid plexus epithelium in fatal diabetic ketoacidosis. Exp Mol Pathol. 2007;83: 65–72. doi: 10.1016/j.yexmp.2007.01.006 17335802 PMC1950467

[73] Hoffman WH, Cudrici CD, Zafranskaia E, Rus H. Complement activation in diabetic ketoacidosis brains. Exp Mol Pathol. 2006;80: 283–288. doi: 10.1016/j.yexmp.2005.12.007 16494864

[74] Serratos IN, Castellanos P, Pastor N, Millán-Pacheco C, Rembao D, Pérez-Montfort R, et al. Modeling the interaction between quinolinate and the receptor for advanced glycation end products (RAGE): relevance for early neuropathological processes. PLoS One. 2015;10: e0120221. doi: 10.1371/journal.pone.0120221 25757085 PMC4354912

[75] Guillemin GJ, Croitoru-Lamoury J, Dormont D, Armati PJ, Brew BJ. Quinolinic acid upregulates chemokine production and chemokine receptor expression in astrocytes. Glia. 2003;41: 371–381. doi: 10.1002/glia.10175 12555204

[76] Jhamandas KH, Boegman RJ, Beninger RJ, Miranda AF, Lipic KA. Excitotoxicity of quinolinic acid: modulation by endogenous antagonists. Neurotox Res. 2000;2: 139–155. doi: 10.1007/BF03033790 16787837

[77] Phillips RS, Iradukunda EC, Hughes T, Bowen JP. Modulation of Enzyme Activity in the Kynurenine Pathway by Kynurenine Monooxygenase Inhibition. Front Mol Biosci. 2019;6: 3. doi: 10.3389/fmolb.2019.00003 30800661 PMC6376250

[78] Giffard RG, Monyer H, Christine CW, Choi DW. Acidosis reduces NMDA receptor activation, glutamate neurotoxicity, and oxygen-glucose deprivation neuronal injury in cortical cultures. Brain Res. 1990;506: 339–342. doi: 10.1016/0006-8993(90)91276-m 1967968

[79] Hoffman WH, Pluta RM, Fisher AQ, Wagner MB, Yanovski JA. Transcranial Doppler ultrasound assessment of intracranial hemodynamics in children with diabetic ketoacidosis. J Clin Ultrasound. 1995;23: 517–523. doi: 10.1002/jcu.1870230903 8537473

[80] Roberts JS, Vavilala MS, Schenkman KA, Shaw D, Martin LD, Lam AM. Cerebral hyperemia and impaired cerebral autoregulation associated with diabetic ketoacidosis in critically ill children. Crit Care Med. 2006;34: 2217–2223. doi: 10.1097/01.CCM.0000227182.51591.21 16763506

[81] Wootton-Gorges SL, Buonocore MH, Kuppermann N, Marcin JP, Barnes PD, Neely EK, et al. Cerebral proton magnetic resonance spectroscopy in children with diabetic ketoacidosis. AJNR Am J Neuroradiol. 2007;28: 895–899. 17494665 PMC8134352

[82] Wootton-Gorges SL, Buonocore MH, Caltagirone RA, Kuppermann N, Glaser NS. Progressive decrease in N-acetylaspartate/Creatine ratio in a teenager with type 1 diabetes and repeated episodes of ketoacidosis without clinically apparent cerebral edema: Evidence for permanent brain injury. AJNR Am J Neuroradiol. 2010;31: 780–781. doi: 10.3174/ajnr.A1829 19926705 PMC7964239

[83] Roe TF, Crawford TO, Huff KR, Costin G, Kaufman FR, Nelson MDJ. Brain infarction in children with diabetic ketoacidosis. J Diabetes Complications. 1996;10: 100–108. doi: 10.1016/1056-8727(94)00058-1 8777328

[84] Muir AB, Quisling RG, Yang MCK, Rosenbloom AL. Cerebral edema in childhood diabetic ketoacidosis: natural history, radiographic findings, and early identification. Diabetes Care. 2004;27: 1541–1546. doi: 10.2337/diacare.27.7.1541 15220225

[85] Durr JA, Hoffman WH, Sklar AH, el Gammal T, Steinhart CM. Correlates of brain edema in uncontrolled IDDM. Diabetes. 1992;41: 627–632. doi: 10.2337/diab.41.5.627 1568533

[86] Glaser N, Barnett P, McCaslin I, Nelson D, Trainor J, Louie J, et al. Risk factors for cerebral edema in children with diabetic ketoacidosis. The Pediatric Emergency Medicine Collaborative Research Committee of the American Academy of Pediatrics. N Engl J Med. 2001;344: 264–269. 11172153 10.1056/NEJM200101253440404

[87] Hoffman WH, Artlett CM, Boodhoo D, Gilliland MGF, Ortiz L, Mulder D, et al. Markers of immune-mediated inflammation in the brains of young adults and adolescents with type 1 diabetes and fatal diabetic ketoacidosis. Is there a difference? Exp Mol Pathol. 2017;102: 505–514. doi: 10.1016/j.yexmp.2017.05.013 28533125

[88] Magnusson KR, Brim BL, Das SR. Selective Vulnerabilities of N-methyl-D-aspartate (NMDA) Receptors During Brain Aging. Front Aging Neurosci. 2010;2: 11. doi: 10.3389/fnagi.2010.00011 20552049 PMC2874396

[89] Moroni F. Tryptophan metabolism and brain function: focus on kynurenine and other indole metabolites. Eur J Pharmacol. 1999;375: 87–100. doi: 10.1016/s0014-2999(99)00196-x 10443567

[90] Chmiel-Perzyńska I, Perzyński A, Urbańska EM. Experimental diabetes mellitus type 1 increases hippocampal content of kynurenic acid in rats. Pharmacol Rep. 2014;66: 1134–1139. doi: 10.1016/j.pharep.2014.07.014 25443746

[91] Wirthgen E, Hoeflich A, Rebl A, Günther J. Kynurenic Acid: The Janus-Faced Role of an Immunomodulatory Tryptophan Metabolite and Its Link to Pathological Conditions. Front Immunol. 2017;8: 1957. doi: 10.3389/fimmu.2017.01957 29379504 PMC5770815

[92] Darlington LG, Forrest CM, Mackay GM, Smith RA, Smith AJ, Stoy N, et al. On the Biological Importance of the 3-hydroxyanthranilic Acid: Anthranilic Acid Ratio. Int J Tryptophan Res. 2010;3: 51–59. doi: 10.4137/ijtr.s4282 22084587 PMC3195249

[93] Badawy AAB, Dougherty DM. Assessment of the human kynurenine pathway: Comparisons and clinical implications of ethnic and gender differences in plasma tryptophan, kynurenine metabolites, and enzyme expressions at baseline and after acute tryptophan loading and depletion. Int J Tryptophan Res. 2016;9: 31–49. doi: 10.4137/IJTR.S38189 27547036 PMC4981220

[94] Ness RB, Haggerty CL, Harger G, Ferrell R. Differential distribution of allelic variants in cytokine genes among African Americans and White Americans. Am J Epidemiol. 2004;160: 1033–1038. doi: 10.1093/aje/kwh325 15561982

[95] MacKenzie KE, Wiltshire EJ, Gent R, Hirte C, Piotto L, Couper JJ. Folate and vitamin B6 rapidly normalize endothelial dysfunction in children with type 1 diabetes mellitus. Pediatrics. 2006;118: 242–253. doi: 10.1542/peds.2005-2143 16818571

[96] Wang G, Li W, Lu X, Zhao X. Riboflavin alleviates cardiac failure in Type I diabetic cardiomyopathy. Heart Int. 2011;6: e21. doi: 10.4081/hi.2011.e21 22355488 PMC3282438

[97] Ulvik A, Theofylaktopoulou D, Midttun Ø, Nygård O, Eussen SJPM, Ueland PM. Substrate product ratios of enzymes in the kynurenine pathway measured in plasma as indicators of functional vitamin B-6 status. Am J Clin Nutr. 2013;98: 934–940. doi: 10.3945/ajcn.113.064998 24004893

[98] Semenkovich K, Bischoff A, Doty T, Nelson S, Siller AF, Hershey T, et al. Clinical presentation and memory function in youth with type 1 diabetes. Pediatr Diabetes. 2016;17: 492–499. doi: 10.1111/pedi.12314 26377697 PMC4803626

[99] Şişmanlar ŞG, Demirbaş-Çakir E, Karakaya I, Çizmecioğlu F, Yavuz CI, Hatun Ş, et al. Posttraumatic stress symptoms in children diagnosed with type 1 diabetes. Ital J Pediatr. 2012;38: 13. doi: 10.1186/1824-7288-38-13 22537813 PMC3480902

[100] Buchberger B, Huppertz H, Krabbe L, Lux B, Mattivi JT, Siafarikas A. Symptoms of depression and anxiety in youth with type 1 diabetes: A systematic review and meta-analysis. Psychoneuroendocrinology. 2016;70: 70–84. doi: 10.1016/j.psyneuen.2016.04.019 27179232

[101] Rechenberg K, Whittemore R, Holland M, Grey M. General and diabetes-specific stress in adolescents with type 1 diabetes. Diabetes Res Clin Pract. 2017;130: 1–8. doi: 10.1016/j.diabres.2017.05.003 28551480 PMC5608607

[102] Shulman R, Luo J, Shah BR. Mental health visits and low socio-economic status in adolescence are associated with complications of Type 1 diabetes in early adulthood: a population-based cohort study. Diabet Med. 2018;35: 920–928. doi: 10.1111/dme.13633 29608218

[103] Siller AF, Lugar H, Rutlin J, Koller JM, Semenkovich K, White NH, et al. Severity of clinical presentation in youth with type 1 diabetes is associated with differences in brain structure. Pediatr Diabetes. 2017;18: 686–695. doi: 10.1111/pedi.12420 27488913 PMC5290262

[104] Wilkinson Michael; Taub P. Sudden cardiac death in young people with diabetes: An opportunity for prevention. Am Coll Cardiol. 2020.

[105] Song P, Ramprasath T, Wang H, Zou M-H. Abnormal kynurenine pathway of tryptophan catabolism in cardiovascular diseases. Cell Mol Life Sci. 2017;74: 2899–2916. doi: 10.1007/s00018-017-2504-2 28314892 PMC5501999

[106] Yu E, Ruiz-Canela M, Guasch-Ferré M, Zheng Y, Toledo E, Clish CB, et al. Increases in Plasma Tryptophan Are Inversely Associated with Incident Cardiovascular Disease in the Prevención con Dieta Mediterránea (PREDIMED) Study. J Nutr. 2017;147: 314–322. doi: 10.3945/jn.116.241711 28179491 PMC5320398

[107] Colpo GD, Venna VR, McCullough LD, Teixeira AL. Systematic Review on the Involvement of the Kynurenine Pathway in Stroke: Pre-clinical and Clinical Evidence. Frontiers in neurology. 2019. p. 778. doi: 10.3389/fneur.2019.00778 31379727 PMC6659442

[108] Schmidt AM, Yan SD, Yan SF, Stern DM. The multiligand receptor RAGE as a progression factor amplifying immune and inflammatory responses. J Clin Invest. 2001;108: 949–955. doi: 10.1172/JCI14002 11581294 PMC200958

[109] McGee MA, Abdel-Rahman AA. N-Methyl-D-Aspartate Receptor Signaling and Function in Cardiovascular Tissues. J Cardiovasc Pharmacol. 2016;68: 97–105. doi: 10.1097/FJC.0000000000000398 27046337 PMC4980173

[110] Niu J, Gilliland MGF, Jin Z, Kolattukudy PE, Hoffman WH. MCP-1and IL-1β expression in the myocardia of two young patients with Type 1 diabetes mellitus and fatal diabetic ketoacidosis. Exp Mol Pathol. 2014;96: 71–79. doi: 10.1016/j.yexmp.2013.11.001 24246157

